# Supplementary material for: Novel transdisciplinary methodology for cross-sectional analysis of snakebite epidemiology at national scale
Source: PLoS Negl Trop Dis. 2021 Feb 12;15(2):e0009023. doi: 10.1371/journal.pntd.0009023 (PMC7906452; doi:10.1371/journal.pntd.0009023)
Supplement: Attachment — Submitted filename: Response Letter Snake-byte Methodology paper 27Aug.docx [file pntd.0009023.s004.docx]

Dear Professor Faiz, dear Professor Chippaux,

We would like to thank the PLOS NTDs reviewers for their time and valuable comments, which we have addressed as thoroughly as possible in the accompanying new version of the manuscript. Please find below our responses to the reviewers’ comments and a description of the changes done.

Considering the comments of the Reviewer #2 who expected some of the results of the community surveys to be included in this paper, we modified the abstract (lines 55-57 and 71-75) to clarify the specific nature of the paper. We emphasize that the paper focuses on explaining our methodology and not the results from its application.

# Methods:

## Reviewer # 2

*“This paper provides a reasonably valuable discussion of epidemiological methods but presents no outcome data whatsoever - this doesn't reflect the title”.*

- We have slightly modified the title to better reflect the focus on the new methodology:

Novel transdisciplinary methodology for cross-sectional analysis of snakebite epidemiology at national scale

Instead of “Development of a transdisciplinary methodology for cross-sectional analysis of snakebite epidemiology at a national scale”

*“While the answers are therefore Yes to these questions, I think the paper must include the data generated by the methodologies selected by the authors”.*

- In addition to the outcome of the epidemiological methods, which are already described in the paper as sample and cluster sizes, there are three other stages described in the methodology, the random cluster selection, the random household selection, and the survey. The cluster selection is unique and depends on the values and filtering parameters chosen by each potential user of this methodology. Therefore, even if we can attach lists of the clusters selected for each country, that information will not add anything to the goal of the paper. Regarding the household selection, the process is random for every single cluster, so it is not replicable, but as for the clusters, sharing the locations or images of the households selected does not contribute to the body of knowledge.
- In addition, the points would have to be anonymized for ethical reasons, which will mean that the readers would have access to 500 images of random points or a list with more than 25,000 random coordinates.
- Finally, the actual data resulting from the surveys cannot be shared at this stage or in this paper, since it is currently being analysed for several additional publications and it is outside the scope of a methodological paper.

## Reviewer # 3

*“Design Effect: Sample size estimation has one big issue in cluster survey. That is design effect (DE). The investigators have used 1.8 as DE, which may be too small. A DE of at least 3 might have been preferable and 2 would be a constraint value.”*

- As is mentioned in lines 315-316, we would like to underline that the design effect value used in our calculations was eventually set to 2.0. Indeed, in addition to other measures aimed at increasing robustness and precision of the estimates (confidence level of 99% instead of 95% in most studies, high number of clusters = 250, additional margin of 15% for non-response), we finally used a DE = 2, which is indeed preferable over this value of 1.8 (even 1.5-1.7 used in MICS and DHS).
- Modification: none. (already addressed in line 315-316).

# Results:

## Reviewer # 1

*“The authors need to be more explicit on why the survey in Cameroon covered most of the country, while in Nepal covered only the Terai region. An explanation is provided in line 370 and thereafter, but it seems insufficient to this reviewer”*

- We agree and we have now extended our supporting arguments for considering only the Terai region in Nepal.
- Modification: extended supporting arguments for considering only the Terai region is now given in lines 375-379.

*“Why was the minimal estimated incidence for both countries the same (100 cases per 100,000 pop. per year)?”*

- We considered 100 cases per 100,000 pop. per year as a minimal incidence in both countries, because both countries pre-existing smaller/local studies show much higher annual incidences of around 75-200 (in Cameroon) up to 1000 (one study in few districts in Nepal) per 100,000. Therefore, we chose 100/100,000 as a “conservative” incidence allowing possible over-powering, rather than the contrary.
- Modification: this explanation is added in lines 311-314).

*The inclusion of snakebites in domestic animals is a novelty of this approach and goes along the paradigm of One Health. However, the authors need to consider that this type of survey involves several species of domestic animals, which vary depending on the region. How is this heterogeneity in the type of prevalent domestic animals taken into consideration in the methodology?*

- This is an important comment that we had indeed already considered in the manuscript (line 195) with the sentence “Although differences in the number and type of domestic and livestock animals per household across regions in each of our study countries are expected, animals are a fundamental source of income in rural communities, and it could be anticipated that most sampled household in the sampling frame will own domestic animals [40]”. Our methodology aims to ensure the geographical representativeness of the selected households through a random selection of clusters and random geospatial sampling and individual selection of household.
- We did not take into account the potential heterogeneity in the geographic distribution of domestic animals. This is one of the challenges of One Health studies where humans and animals are investigated simultaneously. Randomization can only be applied to one population, therefore in this work, and as stated in the methodology, “the domestic animal sample is incidental to that for humans”. This is a limitation of One Health surveys that cover humans and a range of animal species, and this may lead to some bias in the epidemiological results for the animal population that will need to be addressed during data analysis and discussed in the papers that will report them.
- Modification: To address this concern we have added the following sentence to the study limitations section of the paper on line 572:

"Because the domestic animal sample is incidental to that for humans, animal population characteristics, such as the heterogeneity in the distribution of the animal populations, might not be fully captured. This might lead to some bias and it is a common limitation of surveys conducted with a One Health approach where multiple species, including humans, are sampled."

# Conclusions:

## Reviewer # 2

*“The authors assertion that their multi-disciplinary methodology enables the most robust national estimates of snakebite incidence remains questionable because no data was provided to compare with other data.”*

- We were a bit surprised by the reviewer's comment, as we never wrote in the manuscript that our methodology would provide “the most robust national estimates of snakebite incidence”. Throughout the document we provided arguments to highlight the advantages of previous national studies, and how the inclusion of additional elements enriched their results.
- We also emphasized how the analysis of previous methodologies led us to the methodological choices we present, as well as the likely advantages and limitations of applying our methodology in future studies. However, to tone down a bit the last paragraph of the Conclusion, which goes in the direction of the reviewer's comment, we changed (in lines 586-587) “The results and experiences from the application of this methodology will bring snakebite…” by “We hope this methodology will contribute to bring snakebite…”.
- We have addressed the reviewer’s recurrent request for the survey data in previous points.
- Modification: Same changes in lines 55-57 and 71-75, as described previously.

# Editorial and data presentation modifications:

## Reviewer # 2

*“Re the livestock/animal snakebite estimates - it would be interesting to understand how the authors could confidently identify that the animals had been subjected to snakebite as opposed to similar pathologies exerted from other causes (spider/scorpion bites; other pathogens).”*

- We agree that snakebite diagnosis in animals could be challenging. To address this diagnostic issue, we had included in the questionnaire the following question:

How did you conclude that the animal was bitten by a snake?
a.            Because you, or another person, witnessed the bite
b.            Because the animal has been seen interacting with a snake (playing, fighting), a live/dead snake was found close to the animal
c.            Because you found fang marks on the animal
d.            Because you observed specific envenoming signs
e.            Because you found the animal dead
f.             Other _____

- We have also listed specific envenoming signs in animals based on our scoping review on the impact of snakebite on domestic animals ([Bolon et al, 2019](https://www.sciencedirect.com/science/article/pii/S0167587718307001)) and ask the interviewee to select the signs observed that lead him/her to say it was a snakebite. During data analysis, we will categorize animal victims as confirmed, probable or suspected snakebite case based on the answers to these questions.
- Modification: To clarify this in the manuscript, in lines 515-518, we have now added: “Specific questions on the reasons why the interviewee concludes the animal was bitten by a snake and on the clinical signs observed were added in the questionnaire, which helps categorize snakebite in animals as confirmed, probable or suspected during data analysis.”

# Summary and general comments:

## Reviewer # 2

*“See above - provide the data gained from using the selected methodologies.”*

- Same answer as for the previous sections regarding survey data publication.
- Modification: none.

## Reviewer # 3

*“Design Effect: Sample size estimation has one big issue in cluster survey. That is design effect (DE). The investigators have used 1.8 as DE, which may be too small. A DE of at least 3 might have been preferable and 2 would be a constraint value.”*

- This point was already commented upon in the Methods section.
- Modification: none.
